# Modeling Long-Term Facilitation of Respiration During Interval Exercise in Humans

**DOI:** 10.1007/s10439-023-03366-z

**Published:** 2023-09-26

**Authors:** Stanley M. Yamashiro, Takahide Kato, Takaaki Matsumoto

**Affiliations:** 1 Biomedical Engineering Department, University of Southern California, Los Angeles, CA 90089-1111 USA; 2 Department of General Education, National Institute of Technology, Toyota College, Toyota, 471-8525 Japan; 3 School of Health and Sport Sciences, Chukyo University, Toyota, 470-0393 Japan; 4 Biomedical Engineering Department, University of Southern California, 1042 Downey Way, Denny Research Center, Room 140, Los Angeles, CA 90089-1111 USA

**Keywords:** Second-order dynamics plasticity, Exercise, CO_2_ inhalation

## Abstract

Long-term facilitation (LTF) of respiration has been mainly initiated by intermittent hypoxia and resultant chemoreceptor stimulation in humans. Comparable levels of chemoreceptor stimulation can occur in combined exercise and carbon dioxide (CO_2_) inhalation and lead to LTF. This possibility was supported by data collected during combined interval exercise and 3% inhaled CO_2_ in seven normal subjects. These data were further analyzed based on the dynamics involved using mathematical models in this study. Previously estimated peripheral chemoreceptor sensitivity during light exercise (40 W) with air or 3% inhaled CO_2_ approximately doubled resting sensitivity. Ventilation after a delay increased by 17.0 ± 2.48 L/min (*p* < 0.001) during recovery following 45% maximal oxygen uptake ($V_{{{\text{O}}_{2} \max }}$ ) exercise consistent with LTF which exceeded what can be achieved with intermittent hypoxia. Model fitting of the dynamic responses was used to separate neural from chemoreceptor-mediated CO_2_ responses. Exercise of 45% $V_{{{\text{O}}_{2} \max }}$ was followed by ventilation augmentation following initial recovery. Augmentation of LTF developed slowly according to second-order dynamics in accordance with plasticity involving a balance between self-excitatory and self-inhibitory neuronal pools.

## Introduction

Long-term facilitation (LTF) of neural activity has received attention as a means of promoting memory [11] and as applied to respiration to help patients with breathing limitations [20] or avoid sleep apnea [12, 16]. Intermittent strong stimulation of peripheral chemoreceptors with hypoxia or current in animals has been mainly used. Hypoxia has been used in humans and combined with hypercapnia to enhance stimulation [12]. Hypercapnic–hypoxic multiplicative ventilatory interaction has been demonstrated in carotid body stimulation in animals [10]. Variability of hypoxic ventilatory stimulation is well known [22] so alternatives could be useful. Intermittent hypoxia can also lead to undesirable chemoreceptor-induced hypertension depending on the hypoxic level used [6, 21]. In animals, carbon dioxide (CO_2_)-saturated saline has been used to stimulate peripheral chemoreceptors and generate LTF with multiple injections [15]. This allowed studying the neural activity tied to LTF and postulated serotonin release. Strong ventilatory stimulation is possible with combined moderate exercise and 3% CO_2_ inhalation [29]. Ventilation response to three sequential intervals of mild and moderate exercise levels led to a sustained increased ventilation of about 10 L/min which was comparable to intermittent hypoxia [23]. Interval exercise training has been found to be highly effective in improving cardiac ejection fraction in heart failure patients [26]. In interval exercise, the level of exercise is alternated between high and low levels multiple times rather than a single continuous level. It seems possible to also use the interval method to promote LTF. This led to the hypothesis that LTF was caused by enhanced peripheral CO_2_ sensitivity in exercise similar to hypoxia and exercise applied in sequential intervals provided an effective intermittency due to rate sensitivity of neural exercise responses [28]. Sequential exercise intervals were all changed in a step fashion. Prior results in humans involving exercise and CO_2_ inhalation support this possibility during mild exercise and found no significant change in sensitivity at higher exercise levels [24]. Our previous study confirmed these conclusions that peripheral chemosensitivity was enhanced during 40 W exercise, but not significantly increased during 45% maximal oxygen uptake ($V_{{{\text{O}}_{2} \max }}$) exercise [29]. These responses were further analyzed in this study and corrected for changes in partial pressure of end-tidal CO_2_ (${\text{P}}_{{{\text{etCO}}{}_{2}}}$) that occurred during the postulated LTF exercise recovery period to identify the dynamic neural LTF component. The resultant neural LTF response was then interpreted with a second-order dynamics model previously used to interpret phrenic nerve dynamics. LTF responses then differed from the first-order dynamics observed during short term after discharge immediately following chemoreceptor stimulation [14]. Recovery of neural augmentation back to control level fits a single exponential in exercise [28] and up to two exponentials following hypoxia [13].

The purpose of the present work was then to use modeling to define the chemoreceptor and neural roles in mediating LTF following combined interval exercise and mild CO_2_ inhalation.

## LTF Model

**Fig. 1:**
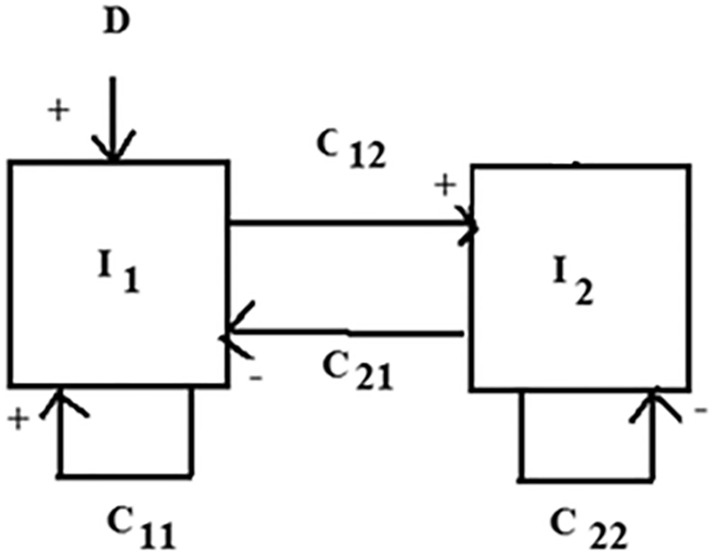
Block diagram of phrenic nerve activity model. Note that the denominator of equation (Bruce et al.) [3] corresponds to a second-order differential equation. The curvilinear shape of the slow gradual increase during long-term facilitation suggests second-order dynamics as will be shown later. First-order or exponential dynamics have a close to linear initial shape

The experimental results of Morris et al. [15] supported the nucleus Raphe obscurus as the site of LTF in response to repeated chemoreceptor stimulation to CO_2_ injections. CO_2_ injection stimulation was then verified to be capable of producing LTF. Two types of neural activity patterns were found in Raphe neurons. One that peaked early following stimulation and subsided and another that slowly increased and was maintained. Integrated phrenic nerve activity was also measured. The major assumption made was treating phrenic activity as an index of minute ventilation. This approach was started by Bruce and von Euler et al. [4] and continues to be useful to the present. Phrenic nerve activity during apneustic breathing in response to CO_2_ stimulation followed a dynamic pattern that can be described by second-order dynamics [3]. Experimental apneusis prevented the normal termination of inspiration and allows the complete inspiratory neural activity pattern to be measured. A block diagram of the model used is shown in Fig. 1. The form of the model is based on a balance between self-excitatory and self-inhibitory neuronal pathways which has been proposed and studied previously in connection with neural respiratory control [8]. The specific details of the current model correspond to Bruce et al. [3] and not Geman and Miller [8]. The differential equations corresponding to this model are as follows:

1 $${t}_{1 }\frac{\mathrm{d}{I}_{1}}{\mathrm{d}t}=-{I}_{1}+ {C}_{11}{I}_{1}-{C}_{21}{I}_{2}+D,$$

2 $${t}_{2} \frac{\mathrm{d}{I}_{2}}{\mathrm{d}t}=-{I}_{2}+ {C}_{12}{I}_{1}-{C}_{22}{I}_{2},$$

where ${I}_{1}$ is instantaneous average activity of the population of excitatory neurons, ${I}_{2}$ is instantaneous average activity of the population of inhibitory neurons.

Parameters *t*_1_ and *t*_2_ correspond to effective neural time constants for build-up and decay of the subpopulations of excitatory and inhibitory neural activity. *D* represents constant tonic (chemical) drive input. *C*_11_, *C*_21_, *C*_12_, and *C*_22_ refer to constant interaction parameters corresponding to interconnections as shown in Fig. 1.

These differential equations can also be described in LaPlace Transform form as transfer functions:

3 $${I}_{1} =\frac{A s+B}{s \left({\left(\frac{s}{{w}_{\mathrm{n}}}\right)}^{2}+2\zeta \left(\frac{s}{{w}_{\mathrm{n}}} \right)+1\right)},$$

4 $${I}_{2}=\frac{{C}_{12}s{I}_{1}}{1+{C}_{22}s\left(\frac{{t}_{2}}{1+{C}_{22} }s+1\right)}.$$

Additional parameters are as follows:

5 $${w}_{\mathrm{n}}^{2}=\frac{{C}_{21}{C}_{12}-({C}_{11}-1)(1+{C}_{22})}{{t}_{1}{t}_{2}},$$

6 $$\zeta =\frac{({t}_{1}\left(1+{C}_{22}\right)-{t}_{2}\left({C}_{11}-1\right)){w}_{\mathrm{n}}}{2},$$

7 $$A=\frac{D{t}_{2}}{{C}_{21}{C}_{12}-({C}_{11}-1)(1+{C}_{22})},$$

8 $$B=\frac{D(1+{C}_{22})}{{C}_{21}{C}_{12}-({C}_{11}-1)(1+{C}_{22})},$$

where ${w}_{\mathrm{n}}$ is natural frequency and *ζ* is damping coefficient of the second-order transfer function. *A* and *B* were defined as shown above to simplify the interpretation of Eqs. (3) and (4).

In fitting phrenic nerve responses to CO_2_, the *A* parameter was set to zero, or assumed to be negligibly small, and *B* was mainly used [3]. Based on this prior result, phrenic peak activity was fitted with a second-order model with three parameters: *B*, *w*_n_, and *ζ*. The *I*_2_ activity is a function of the derivative of *I*_1_ so can be interpreted as a rate sensitivity. Raphe neurons do show a rate sensitivity in the initial rise to a peak followed by a rapid decay [15] matching this model prediction. It must be noted that the main point of the model derivation was the second-order transfer function form as given by Eq. (3). This result justified the second-order description of phrenic and ventilation responses.

## Experimental Methods

### Subjects

The experimental methods have been previously described [9]. Seven healthy, active males with no history of cardiorespiratory diseases volunteered to participate in the present study. Their physical characteristics were as follows: age, height, and weight were 21.7 ± 0.5 years and 171.6 ± 7.4 cm, and 64.5 ± 4.7 kg, respectively. Informed consent was obtained from each subject after a full explanation of the experimental procedure as well as its risks was provided. The experimental protocol was approved by the Human Subjects Committee at the Chukyo University Graduate School of Health and Sport Sciences.

### Exercise Protocol

Each subject performed a $V_{{{\text{O}}_{2} \max }}$ test for deciding the exercise intensity of constant workload exercise (CWE). Exercise was conducted using an electrically braked cycle ergometer (AEROBIKE75XL; Combi Wellness, Tokyo, Japan); the workload was set at 40 W at the beginning of the test and increased by 20 W every minute until exhaustion. Subjects were instructed to maintain a pedaling rate of 70 revolutions per min (rpm). Thereafter, subjects carried out the CWE at moderate (45% $V_{{{\text{O}}_{2} \max }}$) and heavy (80% $V_{{{\text{O}}_{2} \max }}$) intensity using a cycle ergometer. The experimental protocol followed a five-step process. First, rest for 5 min sitting on the bicycle ergometer. Second, baseline cycling at 40 W for 6 min. Third, light (45% $V_{{{\text{O}}_{2} \max }}$) intensity CWE for 6 min. Fourth, baseline cycling at 40 W for 6 min again. Fifth, heavy (80% $V_{{{\text{O}}_{2} \max }}$) intensity CWE for 6 min. The pedaling rate was 70 rpm during the CWE session and baseline cycling. Each subject performed CWE tests on two occasions under normal barometric pressure, under the following conditions: (1) breathing ambient air (Air) and (2) breathing-enriched CO_2_ gas (CO_2_ 3.03 ± 0.06%; O_2_ 20.99 ± 0.03%; balance N_2_) (3% CO_2_). The subjects were blinded to the inhaled gas composition. During the experiment, respiratory parameters were continuously analyzed using a breath-by-breath gas collection system and analyzed every 30 s using an automatic gas analyzer (RM300; Minato Medical Science, Osaka, Japan). In the present study, recovery period data during baseline cycling at 40 W performed after light (45% $V_{{{\text{O}}_{2} \max }}$) intensity CWE and before heavy (80% $V_{{{\text{O}}_{2} \max }}$) intensity CWE were used for modeling.

### Statistical Analysis

Statistical comparisons of the change in minute ventilation from baseline used paired *t* tests. Two different baselines were used. The baseline for ventilation augmentation was the initial individually measured minute ventilation at the start of 45% $V_{{{\text{O}}_{2} \max }}$ exercise with or without 3% inhaled CO_2_. The baseline for CO_2_ change correction was the initial measured minute ventilation at the start of 40 W exercise with or without 3% inhaled CO_2_. This baseline was also used for recovery from 45% $V_{{{\text{O}}_{2} \max }}$ exercise during which the inhaled gas level was maintained. The variance of the differences between predicted and measured ventilations during the initial 3% CO_2_ inhalation and 40 W exercise interval was assumed to also apply during recovery from 45% $V_{{{\text{O}}_{2} \max }}$ exercise and maintained second interval of 3% CO_2_ inhalation and 40 W exercise.

## Results

### Integrated Phrenic Response

**Fig. 2:**
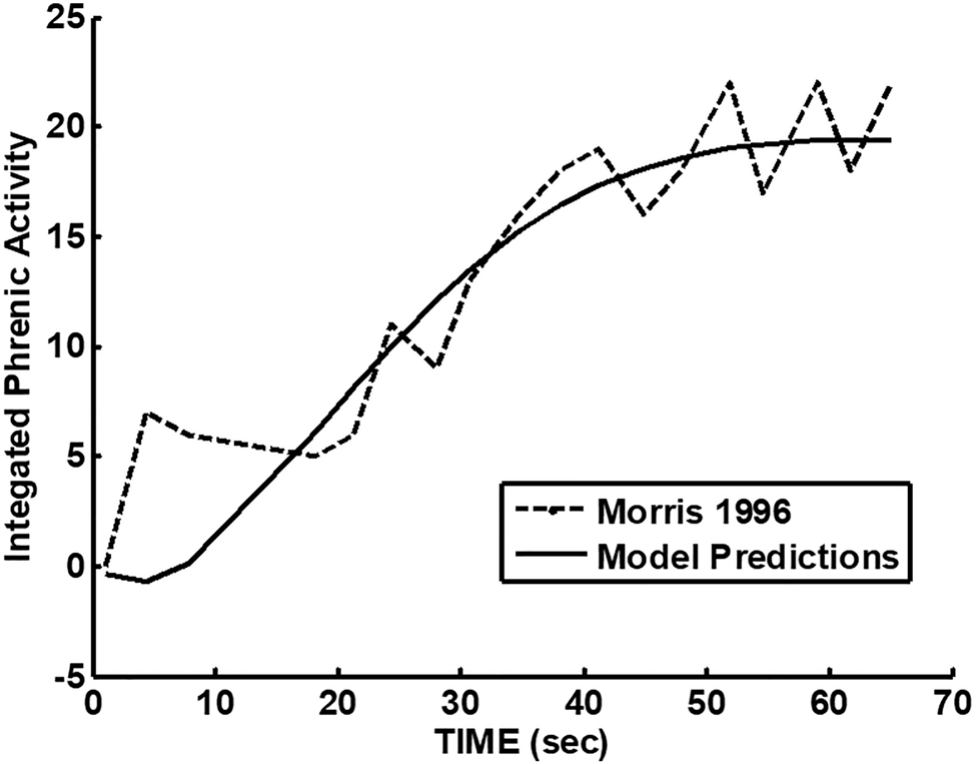
Long-term facilitation model predictions compared to peak integrated phrenic data (*w*_n_ = 0.075 rad/s, *ζ* = 0.7, step = 18.5). Time zero is when stimulation ended

The experimental peak-integrated phrenic activity response following multiple CO_2_ stimulations (Fig. 5) of [15] leading to LTF augmentation was reproduced and compared to second-order model predictions in Fig. 2. This response followed several stimulations that failed to lead to continued augmentation so supported the existence of an apparent threshold for LTF augmentation. Dynamics does fit a second-order model using three parameters. Note that the initial upward curvature of the transient response is not consistent with a first-order exponential response. This is significant because this differs from both respiratory after discharge [14] and post-exercise respiratory transients [28] which show decaying exponential responses. This apparent threshold most likely represents where excitatory exceeded inhibitory neural drives and overcame an augmentation balance. Thus, multiple LTF levels should be possible.

### Human LTF Response

**Fig. 3:**
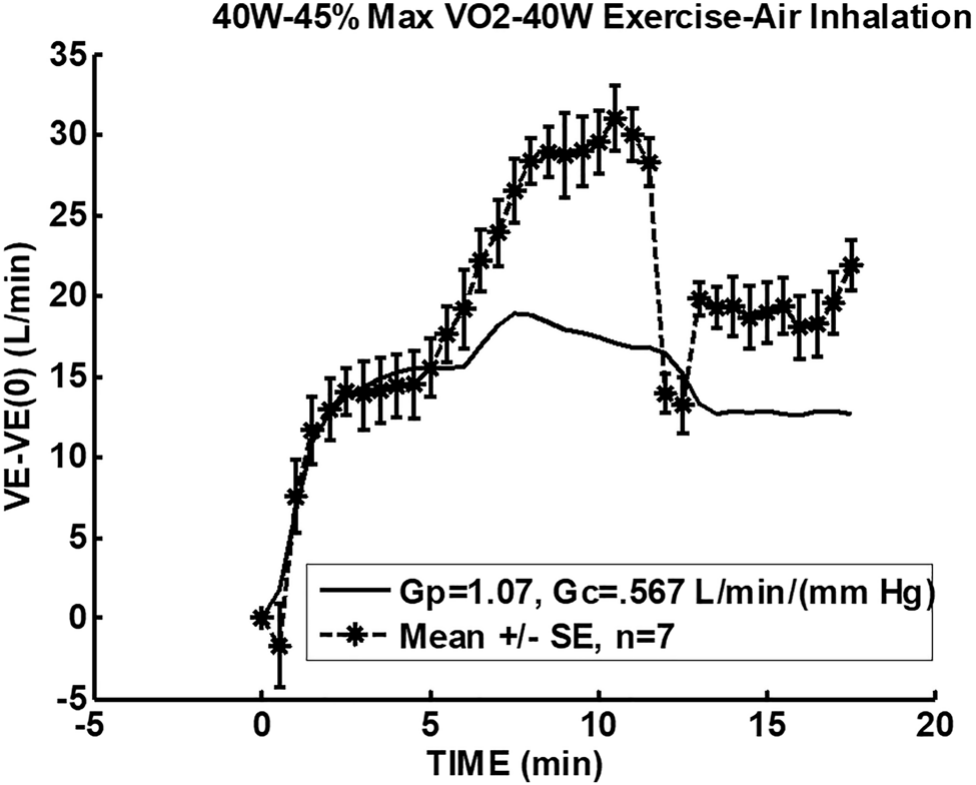
Chemoreceptor model predictions compared to group data during air breathing. Subjects carried out three intermittent level change exercises: rest to 40 W, 40 W to 45% maximal oxygen uptake ($V_{{{\text{O}}_{2} \max }}$), and 45% $V_{{{\text{O}}_{2} \max }}$ to 40 W. *VE* minute ventilation, *G*_*p*_ peripheral gain in (L/min)/mmHg, *G*_*c*_ central gain in (L/min)/mmHg

**Fig. 4:**
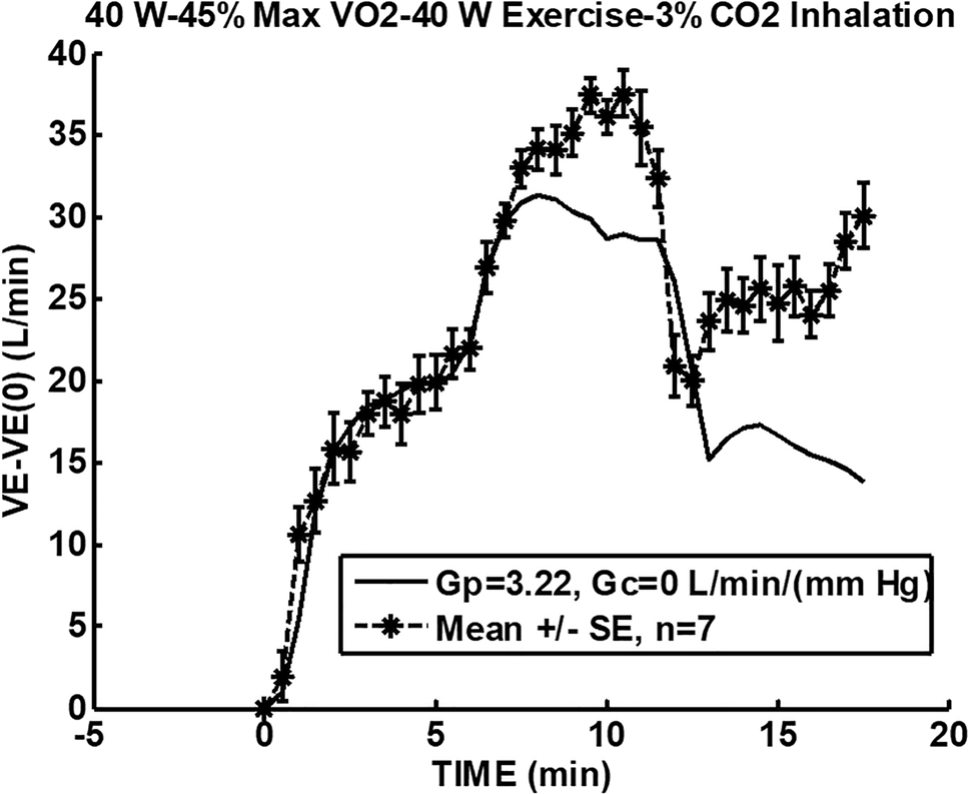
Chemoreceptor model predictions compared to group data during 3% CO_2_ inhalation. Subjects carried out three intermittent level change exercises: rest to 40 W, 40 W to 45% maximal oxygen uptake ($V_{{{\text{O}}_{2} \max }}$), and 45% $V_{{{\text{O}}_{2} \max }}$ to 40 W. *VE* minute ventilation, *G*_*p*_ peripheral gain in (L/min)/mmHg, *G*_*c*_ central gain in (L/min)/mmHg. The standard deviation (SD) for the CO_2_ fit for 40 W exercise + CO_2_ = 1.73 L/min, standard error (SE) = 1.73/sqrt (7) = 0.654. The SD of the final ventilation = 6.32 L/min. By adding the variance of the CO_2_ fit, total combined variance = 6.32^2^ + 1.73^2^, total combined SD = 6.55 L/min, SE = 6.55/sqrt (7) = 2.48 L/min for the final ventilation corrected for CO_2_ changes from baseline

**Fig. 5:**
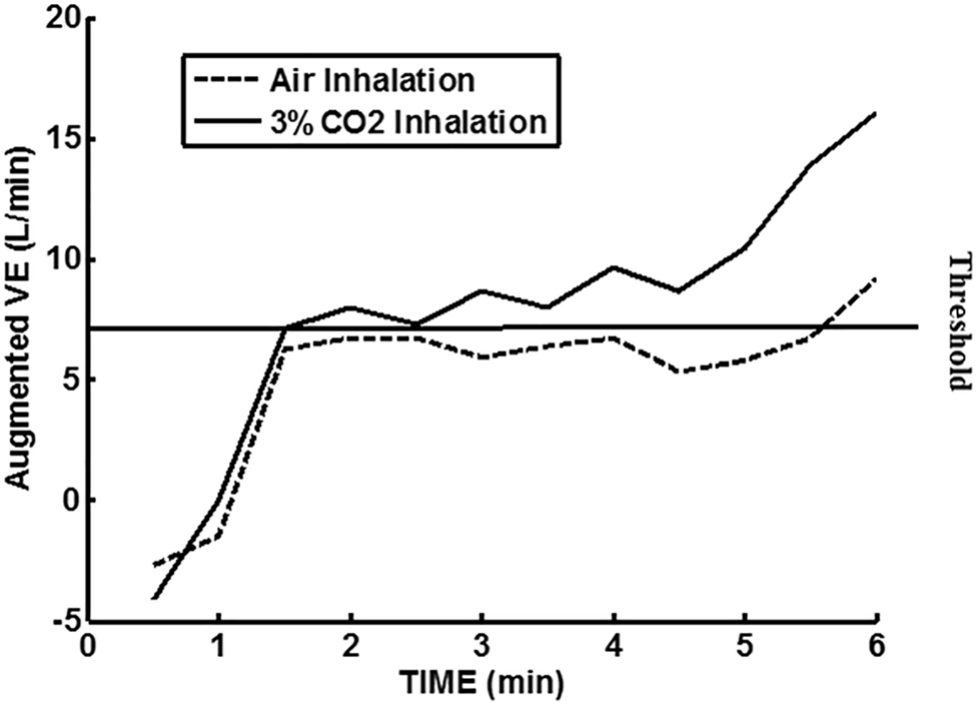
Estimated neurally mediated ventilation augmentation after removal of chemoreceptor-mediated contribution. *VE* minute ventilation. Maximum augmentation estimated was 17.0 ± 2.48 L/min over control level. *p* < 0.001 (see caption for Fig. 4)

**Fig. 6:**
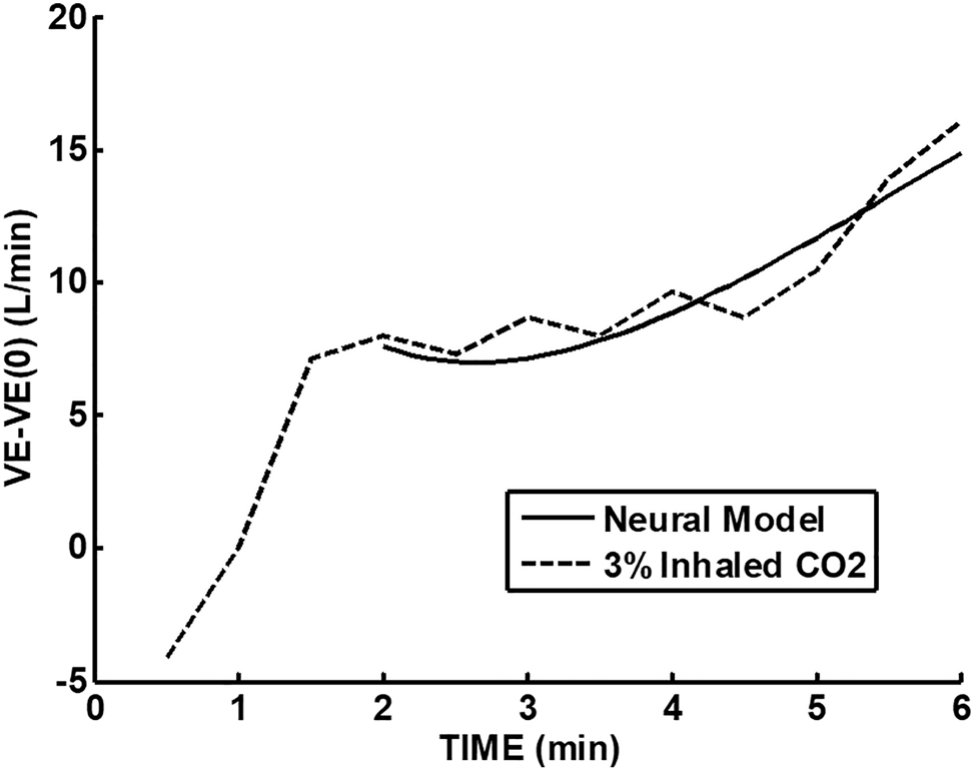
Neural model predictions compared to estimated ventilation from Fig. 4 (*w*_n_ = 0.375, *ζ* = 0.7, *B* = 9, *A* = 0, baseline = 7.6 L/min, start *τ* = 2). *VE* minute ventilation

**Fig. 7:**
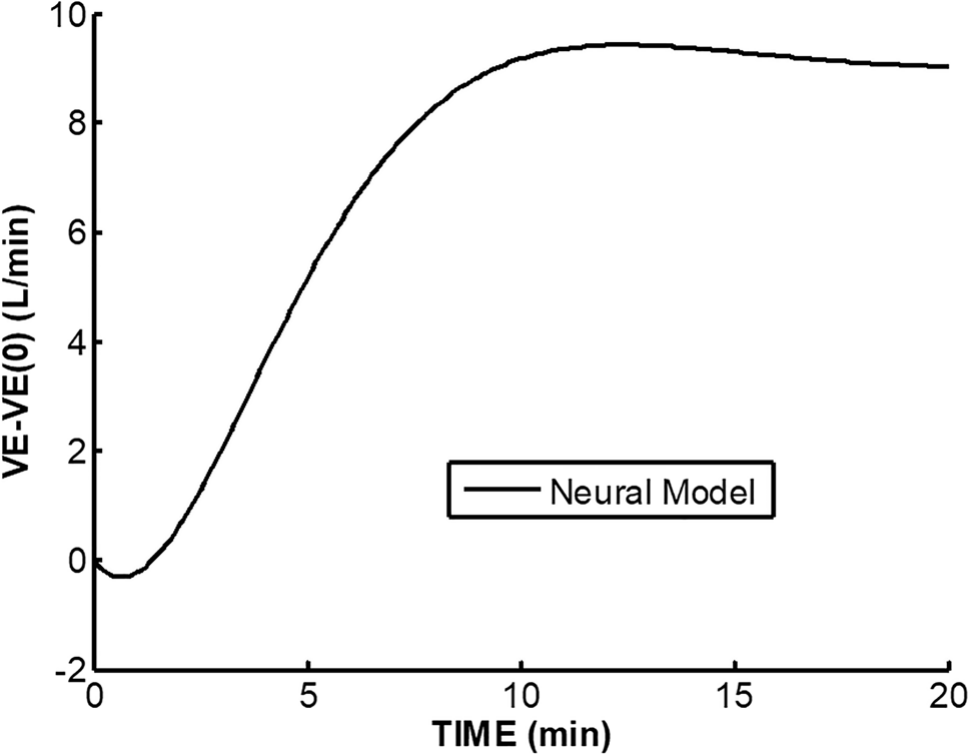
Predicted neural augmentation past observation period. *VE* minute ventilation

In Fig. 3, the model of Bellville et al. [2] with central and peripheral chemoreceptor first-order dynamics (see “Appendix”) was used to fit group averaged (*n* = 7) ventilation change from baseline responses to the measured $P_{{{\text{etCO}}_{2} }}$ changes from baseline during air breathing. Central and peripheral chemoreceptor gains were estimated from least squares fitting using MATLAB using fminsearch as described in our previous study [29]. Only data for the initial 40 W exercise were used for parameter estimation and predictions for all exercises were based on these estimates. In this way, chemoreceptor contributions for mild exercise could be estimated and removed from the final recovery period which also involved the same level of mild exercise. Figure 4 shows a similar fit during 3% CO_2_ inhalation. The group averaged ventilation response after removal of the predicted chemoreceptor contribution is shown in Fig. 5. The control level for ventilatory augmentation was shifted to the beginning of the 45% $V_{{{\text{O}}_{2} \max }}$ exercise period. Also shown in Fig. 5 is a visually estimated threshold which had to be exceeded before additional ventilation increases was observed for both air and 3% CO_2_ inhalations. The threshold was chosen as the point where a rise in ventilation could just be detected. This threshold appeared to represent a balance between excitatory and inhibitory neural drives. The ventilation augmentation during recovery during 3% CO_2_ inhalation was fitted with a second-order response as shown in Fig. 6. The estimated final ventilation level shown in Fig. 5 was 17.0 ± 2.48 L/min SE (*p* < 0.001) above control level. The variance was estimated by adding variances of the group measurement shown in Fig. 4 and the variance of the difference between predicted and measured responses to CO_2_ during 40 W exercise which is also shown in Fig. 4. The collected data end after 6 min, but Fig. 7 shows the subsequent predictions which estimate the final steady-state level of about 9 L/min above the initial augmentation. No data were collected to support this steady-state value, so it merely suggests a model-predicted limit to augmentation. However, animal data shown in Fig. 2 do support steady-state predictions. The initial response clearly shows an initial upward curvature which can only be predicted using a second-order rather than a first-order model. Thus, this pattern of augmentation differs from the decaying exponential pattern observed in post-hyperventilation after discharge [19] and after exercise [28] in humans. Combined interval exercise and inhaled CO_2_ appear to be a promising method to effectively promote LTF while avoiding use of hypoxia and potential complications.

## Discussion

Combined 4% inhaled CO_2_ and heavy exercise has been reported to increase oxygen debt [17]. However, this study also reported that light exercise (45% $V_{{{\text{O}}_{2} \max }}$) did not increase oxygen debt. The significance of this finding was our use of this identical light exercise level. This reference then supports no oxygen debt increase in our recovery responses to 3% CO_2_ and 45% $V_{{{\text{O}}_{2} \max }}$ exercise. The baseline level of exercise used (40 W) was also the same. Thus, what we interpret as LTF responses does not involve recovery to oxygen debt. Intermittent hypercapnia involving 15% inhaled CO_2_ has been reported to lead to neural plasticity of depression rather than stimulation [18]. Raphe neurons were then long-term depressed rather than stimulated by CO_2_ inhalation. The level of inhaled CO_2_ of 15% can lead to a partial pressure in the lungs greater than 100 mmHg. The potential narcotic effect of CO_2_ is well documented. Partial pressures of arterial ${{P}}_{{{\text{CO}}{}_{2}}}$ > 70 mmHg can lead to depression of ventilation [25]. In the Safic et al. study [18], inspection of peak phrenic responses to intermittent inhaled CO_2_ shows a decline after an initial stimulation especially after the first CO_2_ inhalation period. Thus, narcotic effects of CO_2_ appear to be involved in these experiments. In our experiments, a much lower level of inhaled 3% CO_2_ was used with no indication of narcotic effects. The predicted partial pressure in the lungs is 21 mmHg, well below the normal level of 40 mmHg. The difference in model predictions from a first-order model provides a way to distinguish this Raphe neural network-mediated response from short-term potentiation or oxygen debt recovery. A single exponential has been found to describe both responses. A second-order response has an initial upward curved response which is inconsistent with a first-order exponential rise.

LTF is caused by strong stimulation of carotid chemoreceptors by hypoxia and hypercapnia. Intermittent stimulation is most effective and leads to ventilation stimulation that can last for hours [14]. In humans, intermittent hypoxia has been the most common stimulus used. In animal experiments, the use of multiple CO_2_-saturated saline injections was found to be able to stimulate LTF while monitoring Raphe neuron activity which is postulated to lead to serotonin release [15]. Raphe neural activities showed a fast transient and slower sustained activity. The slower delayed sustained activity did not begin until the fast transient activity ended which has been referred to as the “ratchet” mechanism. Thus, a neural mechanism for triggering and a delayed response was suggested. The fast transient response can be interpreted as a rate sensitivity leading to LTF initiation and why intermittency is important in LTF initiation. Peripheral chemoreceptor stimulation by CO_2_ could be integrated and LTF could be delayed until a threshold of LTF generation is reached where excitatory chemoreceptor drives exceed inhibitory drives promoting return to baseline. A balance threshold for LTF is based on the observation that multiple intermittent stimuli are required before a rising phase following initial recovery is observed. The LTF response in animals has been assessed primarily by the peaks of integrated phrenic activity serving as an index of tidal volume [5, 14, 15]. Ventilation changes can be inferred by neural minute activity by including measurement of breathing frequency [14]. Ventilation or neural minute activity changes mirrored tidal changes and a maximum LTF ventilation augmentation of 2.5-folds over control was estimated [14]. This LTF pattern is also supported by the slow development of LTF reported in intermittent hypoxia human trials which uses a lower stimulation level [12, 16, 20, 23]. Exercise has also been previously reported to increase peripheral chemoreceptor stimulation to CO_2_ during mild to moderate exercise [24] similar to hypoxia. Thus, intermittent exercise should also promote LTF. If rate sensitivity is the mechanism for LTF generation then step changes in exercise levels should be sufficient for LTF generation. The current study investigated three exercise level changes: rest to 40 W, 40 W to 45% $V_{{{\text{O}}_{2} \max }}$, and 45% $V_{{{\text{O}}_{2} \max }}$ to 40 W. The significant level of LTF augmentation measured supports this rate sensitivity hypothesis. The third exercise level shift from 45% $V_{{{\text{O}}_{2} \max }}$ to 40 W led to an initial recovery close to the initial baseline ventilation level and was followed by subsequent LTF augmentation. Exercise recovery differs from intermittent hypoxia in that the off-transient does not always show rate sensitivity or short-term potentiation [28]. The delayed second-order dynamic response fit the pattern observed in animals and followed three applied exercise intervals. Air breathing and exercise did appear to also initiate LTF, but it was delayed further and at a lower level of augmentation than 3% CO_2_ inhalation. In the present study, the dynamics of the postulated LTF was focused on. LTF was postulated to have an additive effect on ventilation and to have second-order dynamics. Second-order dynamics was also found to be consistent with a prior animal experiment on LTF [14]. In our previous study [29], a respiratory control dynamic model was fitted to the seven subject CO_2_ response during 40 W exercise. This model was used to correct the ventilation response to the control $P_{{{\text{etCO}}{}_{2}}}$ level to estimate the neural component of LTF augmentation.

The presence of LTF augmentation can be inferred without the use of a model. However, the neural model used was based on previous models hypothesizing a balance of excitatory and inhibitory pools [3, 8] which can be tied to second-order dynamics as shown in Fig. 1 and Eq. (3). Second-order dynamics can be completely described using three parameters as discussed earlier. Animal data previously reported for phrenic nerve activity during LTF was shown in Fig. 2 and could be fitted with a second-order model. This was consistent with earlier phrenic nerve activity modeling during experimental apneusis [3]. The human LTF model applied here as shown in Fig. 6 was the first to propose the use of a second-order model.

Exercise in combination with hypoxia has been previously tried but a 90 s stimulation with 10% inhaled oxygen resulted in short-term potentiation (decay time constant < 30 s) but not LTF [7]. Longer periods of stimulation would probably not be feasible due to possible hypoxic exposure complications.

Human exercise with CO_2_ inhalation and recovery with LTF has not been directly previously reported. However, a previous human exercise study at comparable levels (50% maximum) with $P_{{{\text{etCO}}{}_{2}}}$ controlled 8 mmHg above control level (also comparable) has reported sustained ventilation augmentation of 11.5 L/min after two repetitions [27]. Return to control was reported after 14 days. Observations were interpreted as supporting learning in control of breathing during exercise. LTF may be involved in responses of combined exercise and CO_2_ inhalation in agreement with the present observations.

LTF has received previous attention because it is an example of neural plasticity and memory. The mechanisms involved in the brain appear to involve two different dynamic Raphe neural activity patterns tied to chemoreceptor stimulation [15]. How these patterns interact has not been previously determined. The present modeling study was based on hypothesizing a specific functional interaction leading to a proportional and rate sensitivity with predicted ventilation response which matched the off-transient from three intermittently applied exercises. This is the first dynamic functional interpretation of these neural patterns beyond a block diagram. Functional models of neural control of breathing was started by Clark and von Euler [5] in interpreting rectified and integrated phrenic nerve activity in the same way as ventilation. This approach has stood the test of time and continues to be useful [14, 15]. The current model was based on the same assumed relationship between neural activity and ventilation. The dynamic LTF augmentation pattern has been mainly measured in animals using strong stimulation levels [15]. Human LTF responses have not been previously as well defined most likely due to small magnitude. A recent study reported LTF augmentation due to intermittent hypoxia of only 4–5 L/min [21]. The present dynamic LTF responses were tied to CO_2_ inhalation and exercise rather than hypoxia and was estimated as 17 L/min in augmentation (Fig. 5). Interval exercise and CO_2_ inhalation may be more effective than intermittent hypoxia in promoting human LTF.

The variance of the differences between predicted and measured ventilations during the initial 3% CO_2_ inhalation and 40 W exercise interval was assumed to also apply during recovery from 45% $V_{{{\text{O}}_{2} \max }}$ exercise and maintained second interval of 3% CO_2_ inhalation and 40 W exercise. The measured variances of ventilations measured over seven subjects after 5 min of 3% CO_2_ and 40 W exercise were 28.07 (L/min)^2^ for initial and 31.36 (L/min)^2^ for recovery. The Bartlett test statistic [1] for variance comparison *T* = 0.017 was less than the *χ*^2^ threshold of 3.84 for *p* < 0.05 with 1 degree of freedom and supported the assumption of equal variances.

Separation of chemoreceptor and neural augmentation components was inferred from modeling and not experimentally determined. Chemoreceptor modeling was based on Belleville et al. [2] approach which was validated with data collected in normal and chemoreceptor denervated subjects. Neural modeling based on a second-order dynamic model has been validated by Bruce et al. [3] with animal phrenic nerve activity data. The correspondence of animal phrenic nerve activity data with human ventilation responses was established by Clark and von Euler [5].

The avoidance of hypoxia to promote LTF can be an important advantage because intermittent hypoxia at high enough levels of stimulation can lead to complications due to chemoreceptor-mediated hypertension [6, 21]. Thus, the maximum LTF possible using intermittent hypoxia most likely will be limited by the need to avoid such complications. The combination of interval exercise and mild CO_2_ inhalation leads to comparable ventilation augmentation without the complications of hypoxia.

## Central and Peripheral Chemoreceptor Model

The model used was a simplified version of Bellville et al. [2] It consisted of two differential equations:

Variables: *y*_p_ = ventilation change due to peripheral chemoreceptor = DVE_p_

*y*_c_ = ventilation change due to central chemoreceptor = DVE_c_

DVE = *y*_p_ + *y*_c_ = change in total ventilation from resting level in L/min

DPetCO_2_ = change in $P_{{{\text{etCO}}{}_{2}}}$ from resting level in mmHg

*G*_p_ = peripheral gain in (L/min)/mmHg

*G*_c_ = central gain in (L/min)/mmHg

*T*_p_ = peripheral time constant in min

*T*_c_ = central time constant in min

*T*d*p* = peripheral time delay in min

*T*d*c* = central time delay in min

*x*_in_ = $${\text{D}}P_{{{\text{etCO}}{}_{2}}}$$

9 $$T_{{\text{p}}} {\text{d}}y_{{\text{p}}} /{\text{d}}t = x_{{{\text{in}}}} (t{-}T{\text{d}}p)\;*\;G_{{\text{p}}} {-}y_{{\text{p}}} ,$$

10 $$T_{{\text{c}}} {\text{d}}y_{{\text{c}}} /{\text{d}}t = x_{{{\text{in}}}} (t{-}T{\text{d}}c)\;*\;G_{{\text{c}}} {-}y_{{\text{c}}} .$$

Both of the above equations are first order and can be simulated in MATLAB using transfer functions defined as:

$$H_{{\text{p}}} = tf\left( {\left[ {0\;G_{{\text{p}}} } \right],\;\left[ {T_{{\text{p}}} \;{\text{ }}1} \right],\;'{\text{InputDelay}}',\;T{\text{d}}p} \right)];\;{\text{for}}\;(9),$$

$$H_{{\text{c}}} = tf\left( {\left[ {0\;G_{{\text{c}}} } \right],\;\left[ {T_{{\text{c}}} \;1} \right],\;'{\text{InputDelay'}},\;T{\text{d}}c} \right)];\;{\text{for}}\;(10).$$

Simulation outputs were obtained by:

$$\left[ {y_{{\text{p}}} ,\;\sim } \right] = {\text{lsim}}\left( {H_{{\text{p}}} ,\;x_{{{\text{in}}}} ,\;t,\;0} \right);$$

$$\left[ {y_{{\text{c}}} ,\;\sim } \right] = {\text{lsim}}\left( {H_{{\text{c}}} ,\;x_{{{\text{in}}}} ,\;t,\;0} \right);$$

where *t* is solution time in minutes and 0 sets the initial condition to 0.

All of the temporal parameters were set to normal values as given by Bellville et al. [2] and kept constant throughout. *G*_p_ and *G*_c_ were adjusted to minimize the least squares difference between measured and model-predicted ventilations. The MATLAB function fminsearch was used in combination with the above transfer function models for least square fitting. The fixed temporal constants were as follows:

$$T_{{\text{p}}} = 0.15,\quad {\text{ }}T_{{\text{c}}} = 1.67,\quad {\text{ }}T{\text{d}}p = 0.2,\quad {\text{ and}}\quad {\text{ }}T{\text{d}}c = 0.2\;{\text{all}}\;{\text{in}}\;\min .$$
